# Enhancement of joint flanker effect in intergroup competition

**DOI:** 10.1002/pchj.796

**Published:** 2024-08-21

**Authors:** Yang Zhao, Xucong Hu, Jifan Zhou, Mowei Shen, Haokui Xu

**Affiliations:** ^1^ Department of Psychology and Behavioral Sciences Zhejiang University Hangzhou China; ^2^ Department of Psychology, College of Education Zhejiang University of Technology Hangzhou China

**Keywords:** group competition, group entitativity, joint flanker effect, shared representation, social interaction

## Abstract

Representing the mental state of the partner lays the foundation for successful social interaction. While the representation of group members has been extensively studied, it is unclear how intergroup interactions affect it. In three experiments utilizing the joint flanker task, we found that competition between groups brought about a greater joint flanker effect (Experiment 1). Such phenomenon was not due to competition per se, as competition that occurred between individuals from different groups did not enhance the joint flanker effect (Experiment 2). Using the minimal grouping method to directly manipulate group entitativity, we found that the joint flanker effect was larger when participants perceived the group as being more closely connected; conversely, when they perceived the group as less close, the joint flanker effect was attenuated (Experiment 3). These results suggested that beliefs about the group may be key to how group competition enhanced the joint flanker effect. The potential cognitive mechanisms producing this phenomenon are fully discussed. Overall, our study is the first to explore the impact of intergroup interactions on the joint flanker effect and provides a new perspective on understanding the relationship between within‐group representations and intergroup interactions.

## INTRODUCTION

Humans, as social animals, thrive in communal living, where cooperation among group members is crucial for acquiring sustenance and other resources, as well as for navigating the challenges posed by intergroup competition. To effectively comprehend, coordinate, and collaborate with fellow group members, humans need to represent information about others and plan appropriate interactions accordingly (Echterhoff et al., 2017; Wolf & Tomasello, 2023).

Humans are remarkably good at understanding others' spatial states (Dötsch & Schubö, 2015), goals (Schmitz et al., 2017), actions (Beaurenaut et al., 2021), utility (Jara‐Ettinger et al., 2020), and other information. Such ability is shown since infancy and continues to develop stronger with age (Moll & Meltzoff, 2011; Moll & Tomasello, 2006; Pesowski et al., 2023). Furthermore, many studies have found that processing information about others is likely to be automatic. Even in tasks that are irrelevant to others, humans are still spontaneously influenced by information about others. For instance, in the joint Simon task (Sebanz et al., 2003, 2005), pairs of participants engaged in the task together, each handling one of the two responses. A spatial compatibility effect on reaction times emerged when the participant responsible for another response sat nearby, compared to performing the task individually. This effect was similar to the outcome obtained when a single participant processed both responses simultaneously (traditional Simon task), suggesting the construction of a shared representation of the partner's task, and therefore the effect is also called the social Simon effect. Similar effects were observed in the joint flanker task (Atmaca et al., 2011) and joint spatial numerical association of response codes (SNARC) task (Atmaca et al., 2008), implying that individuals form representations about their group partners. Evidence from perspective‐taking tasks also showed that conflicting information between one's own perspective and another person's perspective interfered with judgments of one's own perspective, suggesting that the other's perspectives were taken into account (Samson et al., 2010). It is important to note that there is a view that the effects revealed by the above tasks do not involve the representation of the state of others (Dolk et al., 2014). Studies have found that similar effects can be observed in the joint Simon task when the partner beside is replaced with a nonsocial object (e.g., a lucky cat ornament; Dolk et al., 2013). One does not claim that such an effect originates from the individual's representation of the internal state of the lucky cat ornament because the ornament has no internal state. There is still debate about the extent to which the social Simon effect (and similar effects) reflects the social nature of cognitive processing. Similar effects in different task contexts may reflect different cognitive processes. The effect of the multi‐person task at least reflects that group membership is a cue that can lead to a representation of the group partner, which is crucial for further maintenance of the social groups.

Representation of others' information often provides benefits to interactions among group members. On the one hand, it promotes inferring and understanding of others' intentions and enhances the efficiency of immediate communication, cooperation, and learning (Frank & Goodman, 2012, 2014; Ho et al., 2022). On the other hand, it helps impression formation and thus brings benefit to peer selection (McEllin et al., 2023). However, in‐group interaction is only one aspect of the group. In real environments, groups usually do not exist in isolation but coexist with other groups. Intergroup interactions are common and have important effects on both the group itself and the group members (De Dreu et al., 2023). It is valuable to explore how intergroup interactions affect one's representation of a group partner and one's within‐group behavior.

The present study aimed to explore the above question by manipulating group interaction, specifically group competition, and measuring its impact on joint flanker tasks. In a traditional flanker task, individuals are required to respond to a central target stimulus while ignoring flanker stimuli that can be either congruent or incongruent. When the flanker stimulus is incongruent with the central target stimulus, reaction times increase significantly compared to both the congruent and baseline conditions, indicating a representation of the flanker stimulus (Eriksen & Eriksen, 1974). The Joint Peer Task (Atmaca et al., 2011) modified this setting by having participants work in pairs, where each participant responded to a set of different targets and did not respond to the partner's targets. When the participant's target was surrounded by the partner's target, it constituted an incongruent condition. Reaction times were significantly increased in the incongruent condition compared to the congruent condition (the flanker stimuli were one's own target) and the baseline condition (the flanker stimuli were not anyone's target). This effect was consistent with the classic Flanker effect, suggesting a representation of the partner's target. The difference in reaction time between the incongruent condition and the other conditions was defined as the joint flanker effect, which reflects interference from the representation of the group partner.

Three experiments were conducted to provide a comprehensive understanding of how intergroup interactions influence the representation of group partners. Experiment 1 aimed to investigate whether intergroup competition enhances the joint flanker effect by comparing joint flanker effects in group‐competition, group‐independent, and single contexts. To distinguish whether such enhancement was due to intergroup competition specifically or general competition effects, in Experiment 2, we constructed the single‐competition contexts in which two participants from different groups competed and investigated whether the joint flanker effect was enhanced in this condition. Experiment 3 further attempted to explore the reasons why the joint flanker effect was enhanced and proposed the hypothesis that intergroup competition changed the perceived group entitativity. To verify this hypothesis, Experiment 3 directly manipulated the perceived group entitativity by adopting the minimum group method and investigated the changes in the joint flanker effect (Yin et al., 2022). If the joint flanker effect was larger when group entitativity was higher, it implied that perceived group entitativity was a key factor affecting the representation of group partners.

## EXPERIMENT 1

### Methods

#### Participants

Forty‐eight undergraduate students (34 females and 14 males, aged 18–25 years) from Zhejiang University participated in Experiment 1. Considering that this study focused on the changes in the joint flanker effect in different intergroup relationships, the sample size followed previous studies using the same task (Atmaca et al., 2011). All participants had normal or corrected‐to‐normal vision, were right‐handed, and used their right index finger to press the key during the experiment. Participants were grouped into teams of four to participate in the experiment at the same time, and these teams were randomly subdivided into groups of two during the course of the experiment. Participants within each team were unacquainted with each other before the experiment. Participants were grouped into teams of four for the experiment. Each participant needed to complete all three conditions: group‐competition, group‐independent, and single. In the group‐competition and group‐independent conditions, participants were randomly divided into pairs within their groups at the beginning of the experiment. All participants were genuine, and none acted as confederates with assigned roles by the experimenter. The two participants within each group were unacquainted with each other before the experiment. This approach ensured that interactions and shared representations were naturally occurring among participants. Upon completion of the experiment, participants received either academic credit or monetary payment. The current experiment and subsequent experiments received approval from the Research Ethics Board of the Department of Psychology and Behavioral Sciences at Zhejiang University and were performed in accordance with the relevant guidelines and regulations. All participants received information sheets about the experimental procedure and signed informed consent forms after learning the purpose and procedure of the experiment. In all three experiments, we reported all the measures, manipulations, and exclusions. All data and research materials were made publicly available via the Open Science Framework and can be accessed at https://osf.io/s9c3a/?view_only=6cfa476d77c84d4db5c9628ff736b495.

#### Design and procedure

**FIGURE 1:**
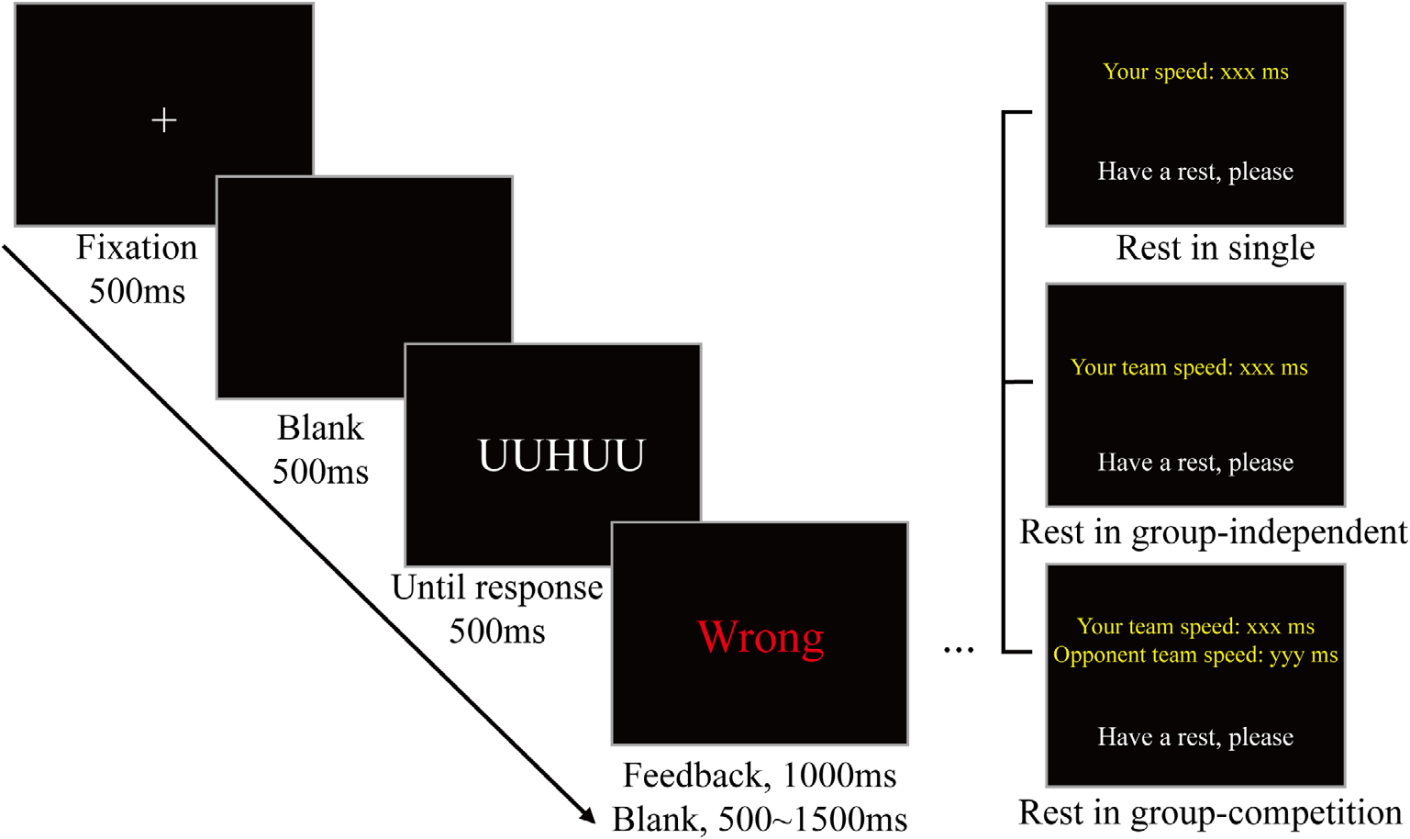
Procedure of the task. Under the three conditions of group‐competition, group‐independent, and single, the content presented in the rest phase was different.

The classic joint flanker task was employed in the experiment. At the beginning of each trial, a white fixation point (red, green, and blue [RGB]: [255, 255, 255], size: 0.41° × 0.41°) was presented on a black background (RGB: [0, 0, 0]) for 500 ms. Following the disappearance of the fixation point, the screen remained blank for 500 ms, and then a stimulus of five white letters (RGB: [255, 255, 255], size: 2.00° × 0.41°) arranged horizontally and closely connected was presented. The central letter in the horizontal row was the target letter (the third one), and the participants were required to press a key corresponding to the identity of the target letter. Subsequent to a response or lack thereof within 1000 ms, the stimulus disappeared, and feedback regarding the accuracy of the response was provided. The next trial began after an interval of 500–1500 ms (Figure 1).

**FIGURE 2:**
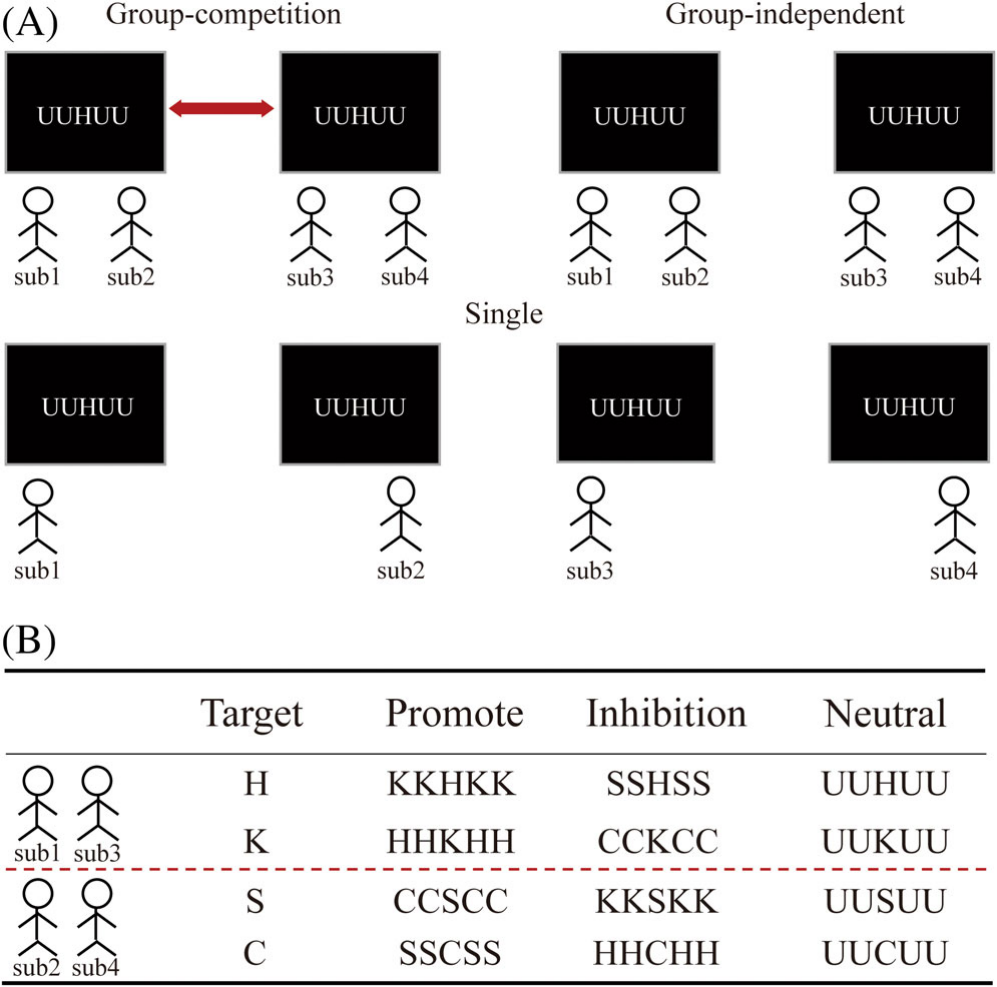
Design of experiment. (A) The illustration of group‐competition, group‐independent and single condition. Each participant took part in all three task context conditions with a fixed partner. (B) The targets and stimuli used in the experiment. Each participant had fixed targets in the three task context conditions.

There were four possible target letters (H, K, S, and C), categorized into two groups (Figure 2B). If the target letter was H or K, the participants were required to press one key on the keyboard (e.g., J); if the target letter was S or C, a different key was to be pressed (e.g., F). The four surrounding letters were identical but distinct from the target letter at the center. Based on the combination of the target letter and the surrounding letters, the stimuli were classified into three types: in the promotion type, the four surrounding letters and the central target letter belonged to the same group (though not the same letters); in the inhibition type, the four surrounding letters were from another group different from the target letter; and in the neutral type, the four surrounding letters were nontarget ones (the letter U was used). Each condition comprised 80 trials, resulting in a total task of 240 trials.

The participants were required to repeat the task in three different contexts: group‐independent, group‐competition, and single contexts. In the group‐independent context, two participants from the same group performed the task together. Each individual was responsible for one set of target letters (one person handled H and K, while the other handled S and C). In the group‐competition context, four participants from two groups performed the task together. Both groups engaged in the identical task as in the group‐independent setting, with the added knowledge that there was competition between the two groups. The group achieving better than 95% accuracy and exhibiting a shorter average reaction time would receive an additional reward. In the single context, the task was exactly the same as in the group‐independent condition, but participants operated individually without another partner beside them. If the presented letter was not their target letter, participants were instructed to wait without responding (Figure 2A).

To enhance the competitive experience, during the rest stage, we displayed the average reaction time of each group. Only the reaction time of the participants' own group was real among the presented reaction times. The “enemy” group's reaction time was randomly generated and updated every 5 s by the program, with a 50% probability of being shorter than that of the participants' own group. In post‐experiment interviews, no participants expressed skepticism regarding the group‐competition aspect of the experiment. Additionally, we also provided participants with their own group's reaction times in the group‐independent and single context to control their visual stimulation and task experience throughout the experiment. The sequence of group contexts was counterbalanced across participants using a Latin square design.

### Results

**FIGURE 3:**
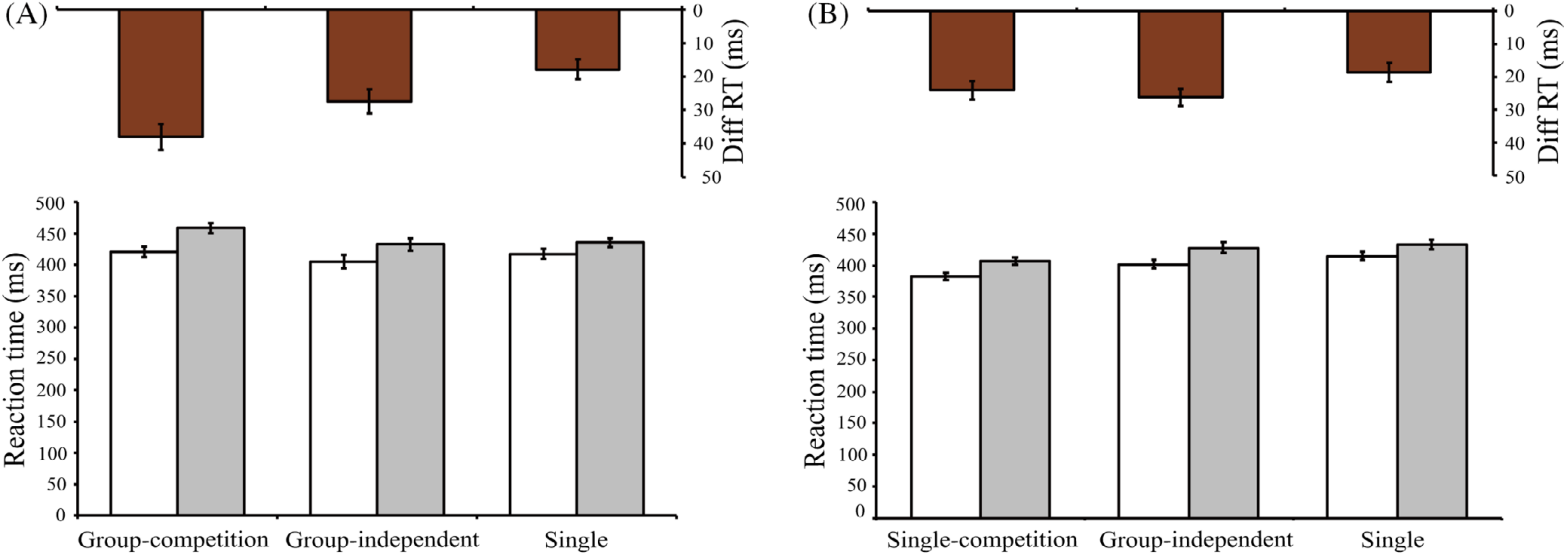
Results from Experiment 1 and Experiment 2. Reaction times (RTs) in baseline (white) and inhibition (gray) conditions. The brown bars on the top represent the difference in RTs between the baseline and inhibition conditions. (A) The difference in RTs (brown bars) in the group‐competition condition was significantly larger than in the group‐independent and single conditions. The group‐independent condition also showed a larger difference in RTs compared to the single condition. (B) The difference in RTs (brown bars) in the group‐independent condition was significantly larger than in the single condition. No significant difference was found between the single‐competition and the other conditions.

Reaction times with correct responses were included in the analysis (Figure 3A). We combined the reaction times in the promote and neutral conditions as the baseline for several reasons. First, both promote and neutral conditions involve nonconflicting stimuli, allowing us to establish a stable baseline by averaging these conditions, thus minimizing variability due to idiosyncratic response differences (Atmaca et al., 2011; Hommel, 2011). Second, this approach is consistent with previous research where neutral conditions were often used to control for general response tendencies while promote conditions control for the influence of compatible stimuli (Eriksen & Eriksen, 1974; Simon & Rudell, 1967). Combining these two conditions helps isolate the specific interference effect introduced by the inhibition condition. We first conducted a 3 (task context) × 2 (stimulus type) repeated measures analysis of variance (ANOVA). The results revealed a significant main effect of stimulus type (*F*[1, 47] = 107.86, *p* < .001, $\eta_{p}^{2}$ = .696), indicating that inhibition stimuli brought about a delay in reaction time. The interaction was significant (*F*[2, 94] = 13.43, *p* < .001, $\eta_{p}^{2}$ = .222), indicating differences in reaction time delay across task context.

To detect the joint flanker effect in group‐independent context, a 2 (task context: group‐independent vs. single) × 2 (stimulus type: inhibition vs. baseline) repeated measures ANOVA was used. The significant interaction (*F*[1, 47] = 6.84, *p* = .012, $\eta_{p}^{2}$ = .127) indicated differences in flanker effects under different task contexts. The reaction time delay caused by the inhibition letters was larger in the group‐independent context than in the single context, consistent with the classical joint flanker effect. This implies that participants in the group‐independent condition represented the target of their partners. A 2 (task context: group competition vs. single) × 2 (stimulus type: inhibition vs. baseline) repeated measures ANOVA was used to detect the joint flanker effect in a group‐competition context. The significant interaction (*F*[1, 47] = 27.79, *p* < .001, $\eta_{p}^{2}$ = .372) showed a similar joint flanker effect, further suggesting that participants in group‐competition condition also represented the target of their partners.

Finally, to compare the effect between group‐competition and group‐independent contexts, we use a 2 (task context: group competition vs. group independent) × 2 (stimulus type: inhibition vs. baseline) repeated measures ANOVA. The significant interaction (*F*[1, 47] = 6.44, *p* = .015, $\eta_{p}^{2}$ = .120) implied a larger joint flanker effect in a group‐competition context than in a group‐independent context. To directly compare the interference caused by the partner's target in different contexts, we computed the difference in reaction times between the inhibition condition and the baseline. The results showed that the reaction time delay in group‐competition context (38 ms) was significantly larger than that in group‐independent (27 ms) and single (18 ms) contexts.

The results of Experiment 1 replicated the classic joint flanker effect and confirmed that individuals spontaneously represent partners' targets unrelated to their own tasks, thereby building a common representation. More importantly, when groups faced intergroup competition, the joint flanker effect was further amplified, implying that the representation of the group partner was affected by the between‐group interaction. However, even though the specific tasks and targets of the participants were exactly the same in the three different task contexts, it was challenging to distinguish whether the enhancement of the joint flanker effect came from the specific influence of intergroup competition or from the general effect of competition situations. Specifically, when individuals were involved in competition, their cognitive control, attention allocation, and emotional state may be affected, rendering their reactions more susceptible to interference. In Experiment 2, we aimed to separate competition and intergroup relationships to verify the aforementioned possibility.

## EXPERIMENT 2

### Methods

#### Participants

Forty‐eight undergraduate students (35 females and 13 males, aged 18–25 years) from Zhejiang University participated in Experiment 2. All participants had normal or corrected‐to‐normal vision, were right‐handed, and used their right index finger to press the key during the experiment. These participants were divided into teams of four to participate in the experiment simultaneously and were randomly subdivided into groups of two during the experiment. Participants in each team did not know each other prior to the experiment. After completing the experiment, all participants received credit or monetary payment.

#### Design and procedure

The design and procedure in Experiment 2 were identical to those in Experiment 1, with the following exception. The group‐competitive context was replaced by the single‐competitive context. In the single‐competition context, two groups of participants still performed the task simultaneously, but the competition no longer occurred at the group level; instead, it occurred at the individual level. Specifically, participants on the same side of the two groups competed, and their individual reaction times were used for comparison. The comparison rules were the same as those in Experiment 1. During the rest phase, the individual's average reaction time and the corresponding opponent's average reaction time were presented on the same side of the screen. The opponent's average reaction time was generated in the same way as in Experiment 1.

### Results

Reaction times with correct responses were included in the analysis (Figure 3B). The baseline reaction time was constructed in the same way as that in Experiment 1. We first conducted a 3 (task context) × 2 (stimulus type) repeated measures ANOVA. The main effect of stimulus type was significant (*F*[1, 47] = 115.59, *p* < .001, $\eta_{p}^{2}$ = .711) and the interaction was also significant (*F*(2, 94) = 3.59, *p* = .031, $\eta_{p}^{2}$ = .071), indicating that inhibition stimuli caused a delay in reaction time, which changed across task contexts.

A 2 (task context: group independent vs. single) × 2 (stimulus type: inhibition vs. baseline) repeated measures ANOVA was used to detect the joint flanker effect in the group‐independent context. The significant interaction (*F*[1, 47] = 8.28, *p* = .006, $\eta_{p}^{2}$ = .150) once again replicated the classical joint flanker effects. However, the 2 (task context: single competition vs. single) × 2 (stimulus type: inhibition vs. baseline) repeated measures ANOVA showed that the interaction effect did not reach significance (*F*[1, 47] = 2.84, *p* = .099, $\eta_{p}^{2}$ = .057). Results from the 2 (task context: single competition vs. group independent) × 2 (stimulus type: inhibition vs. baseline) repeated measures ANOVA also found that the interaction was not significant (*F*[1, 47] = 0.56 *p* = .457, $\eta_{p}^{2}$ = .012). A direct comparison of the interference showed that the reaction time delay in the group‐independent context (26 ms) was significantly larger than that in the single (19 ms) contexts. The reaction time delay in the single‐competition context (24 ms) was not significantly different from those in the other two contexts.

The results of Experiment 2 indicated that when competition occurred between individuals rather than groups, the joint flanker effect was not enhanced but rather weakened. The joint flanker effect even disappeared in the single‐competition context. This meant that the reason for the enhancement in Experiment 1 was not the competition situation but was specific to intergroup competition. We speculated that the root of this phenomenon might be due to the change in individuals' beliefs about the group. Specifically, when competition occurred between groups, the concept of the group was highlighted, and group members were perceived as closer, leading to an enhanced joint flanker effect. We planned to investigate this hypothesis in Experiment 3.

## EXPERIMENT 3

If the enhancement of the joint flanker effect was due to the change of belief about the group, then such enhancement should not be limited to intergroup competition but occur whenever the belief of the group is strengthened. In Experiment 3, we planned to use the minimal group method to manipulate the belief about the group (the perceived entitativity) and detect its impact on the joint flanker effect to further explore the reason for the impact of intergroup interaction. We expected that a similar enhanced joint flanker effect was observed in stronger perceived group entitativity conditions.

### Methods

#### Participants

Ninety‐six undergraduate students (68 females and 28 males, aged 18–25 years) from Zhejiang University participated in Experiment 3. All participants had normal or corrected‐to‐normal vision, were right‐handed, and used right index finger to press the key during the experiment. Participants were divided into teams of four to participate in the experiment simultaneously and were randomly allocated into groups of two during the experiment. Participants in each team did not know each other before the experiment. After completing the experiment, all participants received credit or monetary payment.

#### Design and procedure

When a team of four participants came to join in the experiment, the experimenter would provide bracelets for them. In half of the cases, two participants in the team who were assigned to the same group wore bracelets of the same color (red for one group and blue for another group). We expected that wearing same‐colored bracelets with peers would provide a strong‐grouping signal (strong‐group condition). In the other half of the cases, the two participants on the same side of each group wore bracelets of the same color (both red or both blue), while their partners did not wear bracelets. We expected that wearing the same color bracelet as other group members weakened the grouping message (weak‐group condition).

The task used in Experiment 3 was the same as in the previous experiment, but it only involved the group‐independent and the single context. After finishing the task in the group‐independent context, participants needed to watch a demo. Participants were told that the demo consisted of two people moving, represented as red circles. Participants were then asked to evaluate how closely connected the two agents were in the demo (1 = not at all close, 7 = completely close). The motion of the two circles in the demonstration was actually Brownian motion generated and recorded by a computer program. Participants were then asked to rate how closely they felt connected to their partner during the task. Our aim was to obtain the participants' perception of group entitativity through these two questions (the evaluation on the demo was the baseline).

### Results

We first analyzed the difference in scores between the two evaluation questions to test whether the bracelet manipulation was effective. The independent sample *t*‐test showed that the scores of participants in the strong‐group condition were significantly higher than those in the weak‐group condition (*t*[94] = 2.30, *p* = .024, Cohen's *d* = .468). This demonstrated that the bracelet operation was effective, and wearing the same color bracelet did make the participants feel closer to their partners in the group.

**FIGURE 4:**
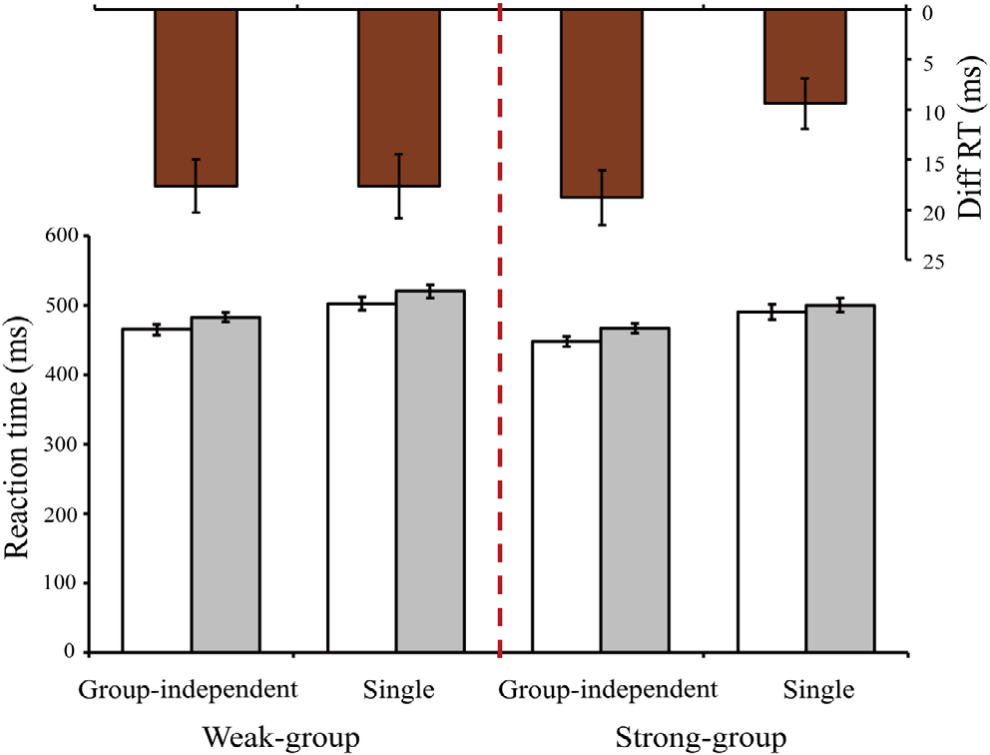
Results in Experiment 3. Reaction times (RTs) in baseline (white) and inhibition (gray) conditions. Their differences were indicated in brown. In the strong group, the “Diff RT” was significantly larger in the group‐independent condition than in the single condition, while the “Diff RT” in the weak group did not show a significant difference.

Reaction times with correct responses were included in the analysis (Figure 4). The baseline reaction time was constructed in the same manner as that in Experiment 1. A 2 (task context: group independent vs. single) × 2 (stimulus type: inhibition vs. baseline) repeated measures ANOVA for the strong‐group condition showed both significant main effects of stimulus type (*F*[1, 47] = 43.40, *p* < .001, $\eta_{p}^{2}$ = .480) and significant interaction (*F*[1, 47] = 7.43, *p* = .009, $\eta_{p}^{2}$ = .137). Direct comparisons of reaction time differences between different stimulus types indicated that reaction time latency was greater in the group‐independent context than in the single context (*t*[47] = 2.73, *p* = .009, Cohen's *d* = .394), which revealed the joint flanker effect. However, the same repeated measures ANOVA for the weak‐group condition showed the significant main effect of stimulus (*F*[1, 47] = 56.36, *p* < .001, $\eta_{p}^{2}$ = .545), while the interaction effect did not reach significance (*F*[1, 47] < .01, *p* = .993, $\eta_{p}^{2}$ < .001). There was no significant difference in reaction time latencies between the group‐independent context and the single context (*t*[47] = −0.01, *p* = .993, Cohen's *d* = .001), suggesting that the joint flanker effect disappeared.

The difference in joint flank effect between the strong‐grouping and weak‐grouping conditions demonstrated that the belief in group entitativity played a key role. When individuals perceived the entitativity of the group to be strong, they were more likely to be disturbed by the group partner's task. Conversely, when the entitativity of the group was considered to be weak, the interference of the group partner's task would be weakened or even disappear.

## GENERAL DISCUSSION

In the joint flanker task, researchers found that when there was a partner nearby, the difference in reaction time between the incongruent condition and baseline condition became larger. This suggests that the presence of a group member may be a cue that leads to the representation of the partner's target. The current study replicated the above results and further found that intergroup competition would enhance the joint flanker effect. Specifically, Experiment 1 created a scenario of intergroup competition. During the competition, the tasks of two participants belonging to the same group were exactly the same as the typical joint flanker task. Only through instructions, letting four subjects perform tasks at the same time, and presenting (false) competition results information, the participants were made to perceive intergroup competition. The results showed that the difference in reaction time between the incongruent condition and the baseline condition increased, which provided preliminary evidence for the interaction between groups affecting the joint flanker effect. Importantly, this increase was not solely attributable to competition itself, as Experiment 2 demonstrated that even when two groups coexisted, competition occurred only between individuals belonging to different groups, the joint flanker effect was not enhanced and, in fact, slightly weakened. This result suggests that the enhancement of the joint flanker effect does not originate from the interaction form of competition but is specifically related to the participants' perception of the group. Experiment 3 provided more direct evidence by manipulating the perceived group entitativity. After using the minimum grouping method to directly weaken the group entitativity, the joint flanker effect disappeared, indicating that perceived group entitativity is an important reason for the change in the joint flanker effect. Collectively, these findings suggest that interactions between groups can alter the joint flanker effect, which may be related to the fact that individuals' perception of the group affects their representation of the group member.

Mammals such as humans evolved to live in social groups, engaging in cooperation with group members to obtain food and other resources while navigating intergroup competition collectively. The importance of groups in the evolutionary process has profoundly influenced humans' distinctive group cognition. On the one hand, studies have revealed that humans can rapidly form social groups based on cues ranging from shared features to common experiences and goals (De Dreu et al., 2023; Henrich & Muthukrishna, 2021; Tomasello & Vaish, 2013). On the other hand, social grouping also restricts attribute inference, behavior prediction, and moral judgment of individuals within the group (Duan et al., 2021; Roberts et al., 2017; Spears, 2021; Yin et al., 2023). Additionally, cognitive activities rooted in social grouping may be automatic and emerge in early childhood (Pesowski et al., 2023; Rhodes & Chalik, 2013). In the task of this study, intergroup competition may serve as a cue to strengthen the perception of the group and make the relationship between individuals and their partners closer. Therefore, individuals are more likely to form a representation of their partners' targets, resulting in an increase in the joint flanker effect.

In addition to representation, changes in the joint flanker effect may also come from changes in attention. In studies such as the joint Simon task and the joint flanker task, a key clue is the presence of the partner. The partner is a visual item with social attributes and may also give social attributes to targets related to the partner. Stimuli with social attributes may attract visual attention. This will cause attention to shift to the partner's target (flanker letter) under incongruent conditions, and reduce attention to one's own task target (central letter), making the difference in reaction time between incongruent condition and baseline condition larger. In our study, the attention attracted by the partner's targets may be further enhanced. This is because, in the context of intergroup competition, the performance of the group is closely related to every group member and is composed of the responses of all members of the group. At this time, although the individual still does not need to respond to the partner's target, the partner's target and response are closely related to the individual. This will lead to more attention being paid to the partner's target, further enhancing the joint flanker effect.

Interestingly, when comparing the group‐independent and single conditions, for example, in Experiment 1, the joint flanker effect seems to be driven by differences in the baseline condition (white) and not by differences in the inhibition condition (gray). This result accords with a similar finding reported by Atmaca et al. (2011), which suggests social facilitation could be a reason for the observed joint flanker effect. People tend to act faster when there are other people present. It is possible that the presence of the partner leads to faster responses to the baseline condition. **However, social facilitation should lead to a decrease in overall reaction time, which is not sufficient to explain the increase in the difference in reaction time between the baseline and incongruent conditions. One possible reason is that the baseline condition is simpler than the incongruent condition, and social facilitation is more likely to occur when the task is easier. However, considering that the task is simple, the difference in difficulty between the baseline (consistent and neutral) conditions and the incongruent condition should be very small. A more reasonable explanation is that the observed joint flanker effect includes the common effect of two factors: the social facilitation effect reduces the overall reaction time, and the representation or attention to the partner's target leads to an increase in the reaction time in the incongruent condition, which ultimately shows an increase in the difference between the incongruent condition and the baseline condition.

It is evident that the representation of others does not necessarily confer advantages to the current task faced by the group. For example, in Experiment 1, when confronted with intergroup competition, the participants were more susceptible to interference from others' tasks, leading to a decrease in the group's response time for the task. So, what benefits does the partner's representation bring to the group? One possible explanation is establishing a common ground for group members, which is the key and foundational element for human communication and cooperation (Wolf & Tomasello, 2023). Human communication and cooperation possess unique characteristics. Not all information needs to be explicitly expressed in words during a conversation, and successful communication can occur without language at times (Faure et al., 2018). Similarly, in cooperation, individuals do not necessarily have to report their every move or closely observe their partner's actions. Cooperation can unfold without detailed planning (Wang et al., 2021). This is possible because individuals can interpret each other's meaning and predict behaviors based on the common ground (Shteynberg et al., 2023). Common ground implies that group members share expectations about the information known to each other, and these expectations rely on shared experiences. As the basis for forming common ground, individuals need to be attentive not only to information relevant to themselves but also to information pertinent to their partners, even if this information may not be useful at the moment.

While not the primary goal of this study, adopting a group perspective on the joint flanker effect may contribute to resolving a major debate in the field: how social is this kind of task? Effects from joint flanker or joint Simon tasks were often conceptualized as a social phenomenon or social cognitive mechanism (e.g., the name “social Simon effect”) due to the involvement of another social agent in the situation. However, some studies argued that the effect was not as inherently social as suggested (Dolk et al., 2014). This perspective was supported by observations of similar effects when a nonsocial object (e.g., a lucky cat ornament) was placed next to human participants in a similar task (Dolk et al., 2013). At this point, claiming that participants spontaneously represented the mental state of an inanimate object is deemed unreasonable because such an inanimate object does not possess a mental state. We propose that if the strength of an effect changes with the manipulation of other social factors in the situation, then it is reasonable to consider the effect and the underlying cognitive mechanisms as social. The joint flanker effect is amplified when there is competition between groups, indicating that the size of the effect is influenced by the social factor of intergroup relations. This provides evidence supporting the social nature of the joint flanker effect. In fact, similar effects would not be expected if one of the participants in our experiment were replaced with an inanimate object. As the inanimate object does not respond, participants would experience interindividual competition akin to the situation in Experiment 2. The results have already shown that interindividual competition does not amplify the joint flanker effect. The specific cognitive mechanism behind the increased difference in reaction time between baseline and incongruent conditions is still unclear. This may be due to shared representations, attention selection, or other social factors, and the cognitive mechanisms may be different when what is next to the participant is human or an inanimate object. Nevertheless, the results of this study indicate that social group is one of the important reasons for the joint flanker effect when two human participants perform the task.

In conclusion, our study, for the first time, provides evidence that intergroup competition enhances the joint flanker effect. The enhancement is linked to beliefs about the group's entitativity. When individuals perceive group members as closer, they are more likely to form representations about group members. Factors such as attention selection and social facilitation are also likely to affect the joint flanker effect. The specific cognitive mechanism behind this effect and how perception of group influences it remain to be further explored in future studies.

## CONFLICT OF INTEREST STATEMENT

Authors declare that they have no competing interests.

### ETHICS STATEMENT

This study was approved by the Research Ethics Board of the Department of Psychology and Behavioral Sciences at the Zhejiang University and was performed in accordance with the relevant guidelines and regulations. All participants received information sheets about the experimental procedure and signed informed consent forms after learning the purpose and procedure of the experiment.

## Data Availability

All data and code have been made publicly available at the OSF and can be accessed at https://osf.io/s9c3a/?view_only=6cfa476d77c84d4db5c9628ff736b495.
